# Endocrine secretory granule production is caused by a lack of REST and intragranular secretory content and accelerated by PROX1

**DOI:** 10.1007/s10735-021-10055-5

**Published:** 2022-01-30

**Authors:** Jun Ishii, Hanako Sato-Yazawa, Korehito Kashiwagi, Kazuhiko Nakadate, Masami Iwamoto, Kakeru Kohno, Chie Miyata-Hiramatsu, Meitetsu Masawa, Masato Onozaki, Shuhei Noda, Tadasuke Miyazawa, Megumi Takagi, Takuya Yazawa

**Affiliations:** 1 Department of Pathology, Dokkyo Medical University School of Medicine and Graduate School of Medicine, Mibu-machi, Tochigi Japan; 2 Education Research Center, Meiji Pharmaceutical University, Kiyose-shi, Tokyo Japan; 3 Department of Pathology, The Jikei University, Minato-ku, Tokyo Japan; 4 Institute of Life Innovation Studies, Toyo University, Itakura-machi, Gunma Japan; 5 Department of Respiratory Medicine, Dokkyo Medical University School of Medicine and Graduate School of Medicine, Mibu-machi, Tochigi Japan; 6 Department of Diagnostic Pathology, Dokkyo Medical University School of Medicine and Graduate School of Medicine, Mibu-machi, Tochigi Japan

**Keywords:** Endocrine secretory granule, REST, PROX1, POMC, Non-neuroendocrine lung cancer

## Abstract

Endocrine secretory granules (ESGs) are morphological characteristics of endocrine/neuroendocrine cells and store peptide hormones/neurotransmitters. ESGs contain prohormones and ESG-related molecules, mainly chromogranin/secretogranin family proteins. However, the precise mechanism of ESG formation has not been elucidated. In this study, we experimentally induced ESGs in the non-neuroendocrine lung cancer cell line H1299. Since repressive element 1 silencing transcription factor (REST) and prospero homeobox 1 (PROX1) are closely associated with the expression of ESG-related molecules, we edited the *REST* gene and/or transfected PROX1 and then performed molecular biology, immunocytochemistry, and electron and immunoelectron microscopy assays to determine whether ESG-related molecules and ESGs were induced in H1299 cells. Although chromogranin/secretogranin family proteins were induced in H1299 cells by knockout of *REST* and the induction was accelerated by the PROX1 transgene, the ESGs could not be defined by electron microscopy. However, a small number of ESGs were detected in the H1299 cells lacking REST and expressing pro-opiomelanocortin (POMC) by electron microscopy. Furthermore, many ESGs were produced in the REST-lacking and PROX1- and POMC-expressing H1299 cells. These findings suggest that a lack of REST and the expression of genes related to ESG content are indispensable for ESG production and that PROX1 accelerates ESG production.

*Trial registration*: Not applicable.

**Supplementary Information:**

The online version contains supplementary material available at 10.1007/s10735-021-10055-5.

## Introduction

Endocrine secretory granules (ESGs) are organelles of endocrine/neuroendocrine cells that selectively store hormones. ESGs can be detected on electron microscopy as approximately 100–400 nm dense-cored spherical structures with a unit membrane (Cross and Mercer 1993). Although ESGs are important morphological markers for the pathological diagnosis of endocrine/neuroendocrine tumours (Graham et al. 1987; Hammond et al. 1998), the mechanism of ESG formation has not been fully elucidated.

ESGs are composed of ESG-related molecules such as chromogranins (CHGs), secretogranins (SCGs), and secretory peptide hormones/neurotransmitters (Brunner et al. 2007; Hickey et al. 2009). The CHG/SCG proteins autonomously aggregate under specific conditions (low pH and high calcium), such as in the trans-Golgi network, and this process involves the aggregation of peptide hormones and is thought to contribute to the selective sorting of secretory contents to ESGs (Chanat et al. 1991; Courel et al. 2008). CHG/SCG family proteins are also precursors for biologically active peptides (Taupenot et al. 2003), and some of them function as helper proteins in the sorting and proteolytic processing of prohormones (Westphal et al. 1999; Mbikey et al. 2001). We and other investigators have reported that the synthesis of most ESG-related molecules is regulated by transrepressive and transactive factors that are specifically expressed in nonendocrine and endocrine cells, respectively. Repressive element 1 (RE1) silencing transcription factor (REST) is known as the former (Schoenherr and Anderson 1995; D'Alessandro et al. 2009; Prada et al. 2011; Kashiwagi et al. 2012; Rovira et al. 2021), and prospero homeobox 1 (PROX1) is known as the latter (Ishii et al. 2016; Saglietti et al. 2019). REST binds to the RE1 sequence in the transcriptional control region of various neuron-specific and ESG-related genes and recruits other transcriptional modulators, such as corepressor of REST (CoREST) and histone deacetylases, resulting in gene inactivation with chromatin aggregation in mainly nonmature neuronal cells (Grimes et al. 2000; Ballas et al. 2005). In contrast, PROX1, which is highly expressed in neuroendocrine cells, binds to a consensus sequence in the transcriptional regulatory cis-element of ESG-related genes and activates the genes (Ishii et al. 2016). When PROX1 expression is experimentally repressed in endocrine cells, the expression of ESG-related genes decreases, thereby decreasing ESGs.

Peptide hormones and neurotransmitters are synthesized as precursors at the rough endoplasmic reticulum (ER), inserted into the ER cisternae, and transported to the Golgi apparatus. Endocrine and neuroendocrine cells have a regulated secretory pathway, and precursors and other regulated secretory pathway proteins are packaged into secretory granules in the trans-Golgi network, processed into active peptides, and secreted in response to specific stimuli (Kim et al. 2006). Overexpression of CHG-A and CHG-B has been shown to induce the formation of ESG-like structures in fibroblasts (Kim et al. 2001; Huh et al. 2003). However, in our previous experiments, although repression of REST or forced expression of PROX1 significantly enhanced the expression of ESG-related genes in non-neuroendocrine lung cancer cells and thyroid cancer cells, we could not identify active ESG biosynthesis (Kashiwagi et al. 2012; Ishii et al. 2016). These results suggested that induction of ESG-related molecules via REST repression alone or PROX1 expression alone was insufficient to activate the biogenesis of ESGs in nonendocrine cells.

The lung has the highest occurrence of neuroendocrine carcinomas among non-neural organs. Approximately 15% of lung cancers are neuroendocrine cancers, and 10–20% of non-neuroendocrine cancers have a neuroendocrine differentiation element (Travis et al. 2004; Fisseler-Eckhoff and Demes 2012; Yazawa 2015). Since lung cancers with neuroendocrine phenotypes have a poor prognosis and since ESGs are electron microscopic hallmarks of neuroendocrine differentiation, a precise understanding of the mechanisms of ESGs in lung cancer cells will promote accurate histopathological diagnosis of lung cancers.

In this study, we conducted *REST* gene editing and PROX1 transgene analysis and attempted to utilize the transgene pro-opiomelanocortin (POMC) as an intragranular molecule to define suitable conditions for producing ESGs using the undifferentiated non-neuroendocrine cancer cell line H1299.

## Materials and methods

### Cell culture

Two human non-neuroendocrine lung carcinoma cell lines (H1299 and TKB5) derived from large cell carcinoma, a human neuroendocrine lung carcinoma cell line (TKB12 and TKB16) derived from small cell carcinoma (Ishii et al. 2013; Endo et al. 1986), a human uterine cervical squamous cell carcinoma cell line (HeLa) and a human embryonic kidney cell line (GP2-293) were used in this study. H1299 and HeLa cells were purchased from the American Type Culture Collection (ATCC, Manassas, VA, USA), and GP2-293 cells were purchased from Clontech (Mountain View, CA, USA). H1299, TKB5, HeLa and GP2-293 cells were cultured in Dulbecco’s modified Eagle’s medium, and TKB12 and TKB16 cells were cultured in RPMI 1640 medium. The media were supplemented with 10% heat-inactivated foetal calf serum, 100 U/ml penicillin, and 100 μg/ml streptomycin. The cells were maintained at 37 ℃ and 5% CO_2_.

### Reverse transcription polymerase chain reaction (RT-PCR) and quantitative RT-PCR (qRT-PCR)

Total RNA was extracted from cultured cells using TRIzol (Invitrogen, Carlsbad, CA, USA). First-strand complementary DNA (cDNA) was synthesized from the total RNA using the SuperScript First-Strand Synthesis System (Invitrogen) according to the manufacturer’s instructions. The resulting cDNA was used as a template for RT-PCR or qRT-PCR, and the specific primer sets are shown in Table 1. For RT-PCR, amplification was performed using GoTaq Green Master Mix (Promega, Madison, WI, USA) and TaKaRa PCR Thermal Cycler Dice Touch (TaKaRa, Shiga, Japan). For qRT-PCR, amplification was performed using a Fast SYBR Green system (Applied Biosystems, Carlsbad, CA, USA) and a StepOnePlus Real-Time PCR System (Applied Biosystems), and the data were obtained from triplicate reactions. The means and standard deviations (SD) of the copy numbers were normalized to the value for ribosomal protein S18 (RPS18) mRNA.

**Table 1:** Sequences of the primers used in this study

Name	Forward primer sequence	Reverse primer sequence	Product size (bp)
RPS18 for qRT-PCR	5′-TTTGCGAGTACTCAACACCAACATC-3′	5′-GAGCATATCTTCGGCCCACAC-3′	89
REST for qRT-PCR	5′-CGCCCATATAAATGTGAACTTTGTC-3′	5′-GGCGGGTTACTTCATGTTGATTAG-3′	145
PROX1 for qRT-PCR	5′-CCCAGCTCCAATATGCTGAAGAC-3′	5′-CACGGAAATTGCTAAACCACTTGA-3′	94
POMC for qRT-PCR	5′-CTACGGCGGTTTCATGACCT-3′	5′-CACTCGCCCTTCTTGTAGGC-3′	96
CHG-A for qRT-PCR	5′-TCCCTGTGAACAGCCCTATGAATAA-3′	5′-AAAGTGTGTCGGAGATGACCTCAA-3′	78
CHG-B for qRT-PCR	5′-CGAGGGGAAGATAGCAGTGAA-3′	5′-CAGCATGTGTTTCCGATCTGG-3′	133
SCG-2 for qRT-PCR	5′-AGCCGAATGGATCAGTGGAA-3′	5′-GATGGTCTAAGTCAGCCTCTGAGAT-3′	84
SCG-3 for qRT-PCR	5′-TCGCTGCCAGGATTTATGAAGA-3′	5′-AGTCCAGCTTGTGGGCTTATTG-3′	194
PROX1 for cloning	5′-GAAGATGGCACAATAACCGTC-3′	5′-ACAAAGTTGAGCAGCGTAAAT-3′	3113
POMC for cloning	5′-GCGAAGGAGGGGAAGAAGAG-3′	5′-CAGGCAGCTTTAAGAGGCTGAT-3′	1083

### Gene Editing

Single-guide RNA (sgRNA) sequences targeting human *REST* (5′-GAGACATATGCGTACTCATTC-3′) were designed using CRISPRdirect (http://crispr.dbcls.jp/). These sequences were separately inserted into pSpCas9 (BB)-2A-Puro (PX459) V2.0 (Addgene plasmid #62988), and the resulting vectors were transfected into cultivated cells with X-tremeGENE 9 DNA Transfection Reagent (Merck, Darmstadt, Germany). After 48 h of transfection, the cells were selected with puromycin (1 μg/ml) for 48 to 72 h. Thereafter, single-cell cloning was performed using On-chip SPiS (On-chip Biotechnologies Co., Ltd., Tokyo, Japan). After growth, genomic DNA was extracted from the cloned cells, and sequencing was performed to confirm *REST* gene knockout.

### Construction of retroviral vectors and transfection

We used a retrovirus-based Retro-X Tet-One Inducible Expression System (pRetroX-TetOne-Puro vector, Clontech) for PROX1 to more easily obtain stable transfectants because PROX1 is associated with unexpected growth inhibitory effects and a pQCXIN retroviral vector (Clontech) for POMC. Complementary DNAs (cDNAs) of PROX1 and POMC were synthesized from DNase-treated total RNA harvested from TKB16 and TKB12 cells, respectively, using the SuperScript First-Strand Synthesis System according to the manufacturer’s instructions (Invitrogen). Then, cDNA was amplified by PCR using a specific primer set (Table 1) and PrimeSTAR GXL DNA Polymerase (TaKaRa). A FLAG-tagged sequence was added to the N-terminus of PROX1 cDNA to enhance the detectability of the PROX1 protein by western blots. After sequencing, the pRetroX-TetOne-Puro vector encoding FLAG-tagged PROX1 cDNA or pQCXIN encoding POMC cDNA was transfected into GP2-293 cells using X-tremeGENE 9 DNA Transfection Reagent (Merck). After 24 h, the conditioned medium was recovered as a viral solution. The desired genes were introduced into cultivated H1299 cells with or without *REST* gene silencing by incubation with the viral solution and 10 mg/ml polybrene (Sigma-Aldrich, St Louis, MO, USA) for 24 h. The cells were selected with puromycin (1 mg/ml) or neomycin (1 mg/ml) for 2 weeks. For induction of PROX1 expression, the transfectants were cultivated in medium with 1 mg/ml doxycycline (Clontech) for 48 h before harvest.

### Western blot analysis

Whole-cell lysates (30 mg protein/lane) were separated by SDS–polyacrylamide gel electrophoresis and transferred onto polyvinylidene difluoride (PVDF) membranes (Merck). The membranes were blocked for 30 min at room temperature with 0.5% skim milk in PBS containing 0.1% (v/v) Tween 20 (PBS-T) and then incubated with a diluted rabbit polyclonal anti-CHG-A antibody (1:250, ab15160, Abcam, Cambridge, UK), mouse monoclonal anti-FLAG antibody (1:1000, F1804, Sigma–Aldrich) or mouse monoclonal anti-ACTH antibody (1:400, ab199007, Abcam) overnight at 4 ℃. An anti-ACTH antibody was used to detect POMC gene products. After three washes for 10 min with PBS-T at room temperature, the membranes were incubated at room temperature for 30 min with a diluted peroxidase-labelled secondary antibody against mouse IgG (1:10,000, NA931V, GE Healthcare, Buckinghamshire, UK) or rabbit IgG (1:5000, NA934V, GE Healthcare). The membranes were then washed three times for 10 min with PBS-T at room temperature, and immunopositive signals were visualized using an enhanced chemiluminescence detection kit (EzWestLumi plus, ATTO). Mouse monoclonal anti-glyceraldehyde-3-phosphate dehydrogenase (GAPDH) (sc-32233, Santa Cruz Biotechnology, Santa Cruz, CA, USA) was used as an internal control.

### Ultrastructural analysis

H1299, REST-deficient H1299 (H1299-RESTKO), PROX1-transfected H1299 (H1299-PROX1), POMC-transfected H1299 (H1299-POMC), REST-deficient and PROX1-transfected H1299 (H1299-RESTKO-PROX1), REST-deficient and POMC-transfected H1299 (H1299-RESTKO-POMC), PROX1- and POMC-transfected H1299 (H1299-PROX1-POMC), and REST-deficient PROX1- and POMC-transfected H1299 (H1299-RESTKO-PROX1-POMC) cells were used. Cultured cells were pelleted and fixed with 2.5% glutaraldehyde in 0.1 mol/l phosphate buffer (pH 7.3) at 4 ℃ overnight, postfixed in 1% osmium tetroxide for 1.5 h, washed twice with distilled water and dehydrated through an ascending series of alcohols. The cell pellet was filled with a 1:1 solution of 100% ethanol and propylene oxide for 20 min, propylene oxide for 20 min twice, a 1:1 solution of propylene oxide and epoxy resin for 2 h, and epoxy resin overnight and then polymerized at 35 ℃ for 8 h, 45 ℃ for 8 h, and 60 ℃ for 48 h. Semithin sections were cut from the resin-embedded blocks, stained with toluidine blue, and used for light microscopy orientation. Ultrathin (70 nm) sections were cut from selected areas, mounted on coated copper grids, stained with uranyl acetate and lead citrate, and subsequently examined under transmission electron microscopy (JEM 1010 electron microscope, JEOL, Tokyo, Japan) at 80 kV.

### Immunofluorescence microscopic analysis

H1299, H1299-RESTKO-PROX1, H1299-RESTKO-POMC, and H1299-RESTKO-PROX1-POMC cell pellets were washed with PBS twice and fixed with 10% buffered formalin solution. The pellets were embedded in paraffin wax and sliced at a thickness of 4 μm for immunofluorescence assays. After microwave treatment for antigen retrieval, the section-mounted slides were incubated with a mixture of a rabbit polyclonal anti-CHG-A antibody (1:100, ab15160, Abcam or 1:1, 412751, Nichirei Bioscience, Inc., Tokyo, Japan) and a mouse monoclonal anti-ACTH antibody (1:400, ab199007, Abcam) overnight at 4 ℃. After being thoroughly washed with 15 mmol/l phosphate-buffered saline (PBS) (pH 7.4), the slides were incubated with a mixture of an Alexa Fluor 488-labelled anti-mouse IgG secondary antibody (1:1000, A32723, Thermo Fisher Scientific, Waltham, MA, USA) and an Alexa Fluor 555-labelled anti-rabbit IgG secondary antibody (1:1000, A32732, Thermo Fisher) for 1 h at room temperature. After thorough washes with PBS, the nuclei were counterstained with 4′6′-diamidino-2-phenylindole (DAPI). Analyses were performed using a confocal microscope (Zeiss LSM 710, Munich, Germany). Based on the captured fluorescence image, the number of colocalization signals of Alexa Fluor 488 and Alexa Fluor 555 was measured using ImageJ software (National Institutes of Health).

### Immunoelectron microscopy analysis

For immunogold labelling, cultured cells were pelleted and fixed with 4% paraformaldehyde and 0.1% glutaraldehyde in PBS for 1 h, dehydrated with a graded series of ethanol at 4 ℃ and embedded in Lowicryl K4M. The resins were polymerized under ultraviolet light for 1 day at room temperature. Ultrathin Sects. (70 nm) were sliced, treated with 10% normal goat serum for 30 min at room temperature, incubated overnight with an anti-CHG-A antibody (1:400, ab15160, Abcam) and incubated with an anti-ACTH antibody (1:200, ab199007, Abcam) for 2 h at room temperature. After washing with PBS, the sections were incubated with goat anti-mouse secondary IgGs coupled to 15 nm gold particles (1:200, BBI Solutions, Cardiff, UK) and goat anti-rabbit secondary IgGs coupled to 5 nm gold (1:200, BBI Solutions) for 2 h at room temperature and then washed with PBS. After drying, the sections were stained with uranyl acetate and lead citrate. The ultrathin sections were examined by transmission electron microscopy, and the images were captured using the CCD camera system.

## Results

### REST gene editing and PROX1 transgene in H1299 cells

**Fig. 1:**
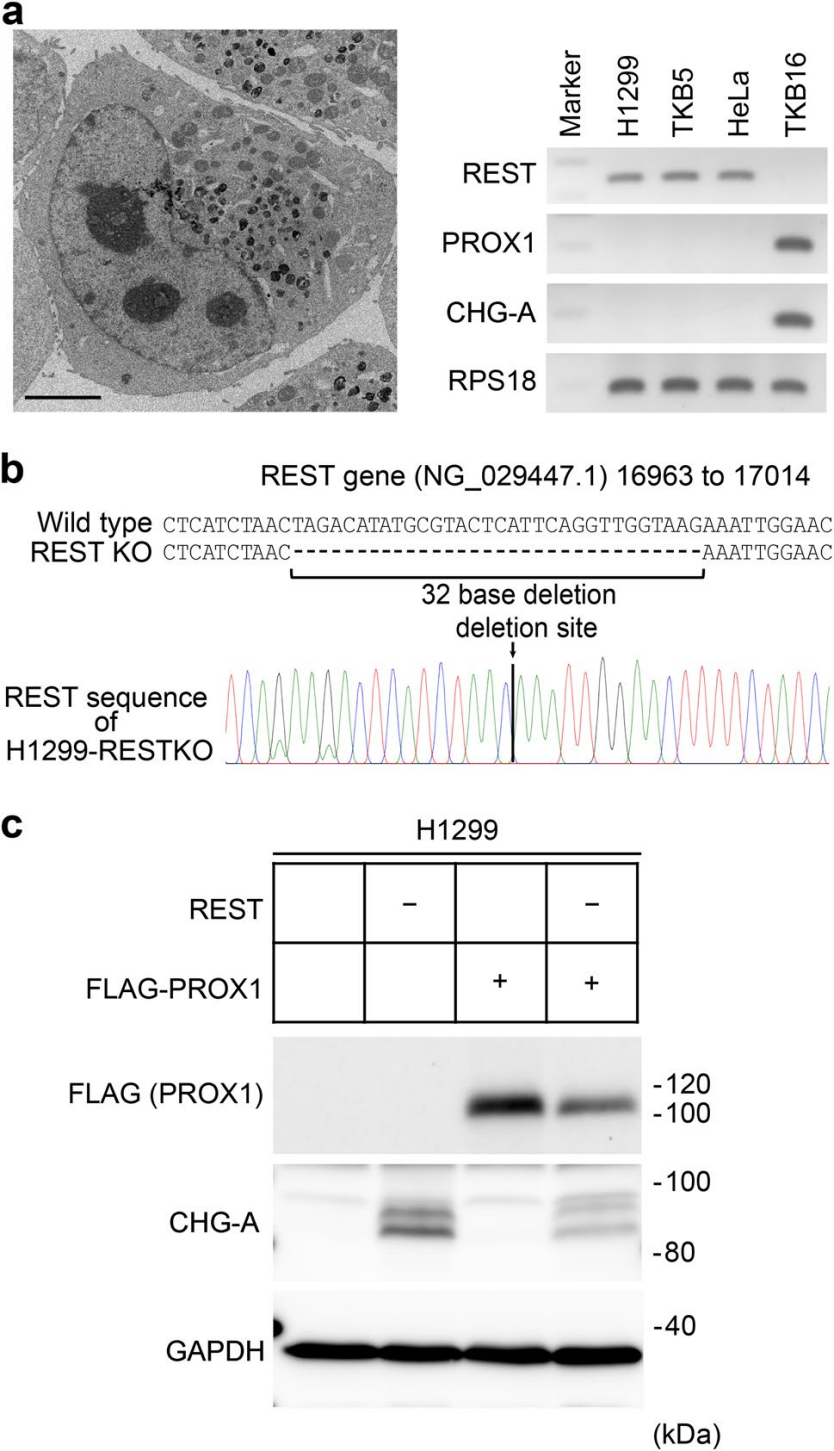
Establishment of *REST* gene-edited and/or PROX1-transfected H1299 cells. **a** (Left) Transmission electron micrograph of H1299 cells. The non-neuroendocrine lung cancer cell line H1299 does not express endocrine secretory granules (ESGs). The large globular structures with high electron density found in cells are secondary lysosomes. Scale bar, 5 μm. (Right) RT-PCR analyses of repressive element 1 silencing transcription factor (REST), prospero homeobox 1 (PROX1) and chromogranin A (CHG-A) in the lung cancer cell lines H1299 (large cell carcinoma), TKB5 (large cell carcinoma), and TKB16 (small cell carcinoma) and HeLa cells. H1299 cells, as well as TKB5 and HeLa cells, expressed REST but did not express PROX1 or CHG-A. PROX1 and CHG-A were expressed in the small cell carcinoma cell line TKB16. Ribosomal protein S18 (RPS18) served as an internal control. Ethidium bromide-stained gel, reverse images. **b** A schematic diagram of the *REST* gene editing status in H1299 cells. In *REST* knockout H1299 cells, the homozygous deletion of 32 bases in the *REST* gene (NG_029447.1, 16963 to 17014) was confirmed by Sanger sequencing. **c** Western blot analysis of FLAG-tagged PROX1 and CHG-A in the H1299 transfectants. FLAG signals were found in the FLAG-tagged PROX1-transfected H1299 cells. CHG-A was induced in the *REST*-deficient H1299 cells. GAPDH served as an internal control. "+" indicates that a specific gene was transfected, and "−" indicates that a specific gene was knocked out

First, we examined whether H1299 cells were suitable for ESG formation experiments. Electron microscopic examination confirmed that H1299 cells did not have ESGs (Fig. 1a, left). Subsequently, the expression of REST, PROX1, and CHG-A in the non-neuroendocrine lung cancer cell lines H1299 and TKB5, the uterine cervical cancer cell line HeLa, and the neuroendocrine lung cancer cell line TKB16 was analysed by RT-PCR. REST expression was found, but no PROX1 or CHG-A signals were observed in the H1299 cells, TKB5 cells or HeLa cells (Fig. 1a, right). However, PROX1 and CHG-A signals were found, and no REST signal was observed in the neuroendocrine lung cancer cell line TKB16 (Fig. 1a, right). These results confirmed that H1299 is a non-neuroendocrine lung cancer cell line that was suitable for this study.

Next, we established H1299-RESTKO, H1299-PROX1, and H1299-RESTKO-PROX1 cells. Knockout of the *REST* gene was confirmed by DNA sequencing (Fig. 1b). In the H1299-RESTKO cells, 32 bases of the *REST* gene sequence (16973 to 17004, sequence ID: NG_029447.1) were homozygously deleted. This deletion site corresponds to part of the 3′ end of exon 3 and to part of the 5′ end of intron 3 of the *REST* gene and causes a frame shift of exon 3, resulting in the appearance of a stop codon after one-third of the REST protein and thereby impairing its function. FLAG tag-added PROX1 (FLAG-PROX1) cDNA was introduced into the H1299 and H1299-RESTKO cells, and the expression of FLAG-PROX1 was confirmed by western blotting (Fig. 1d). CHG-A was not induced in the H1299-PROX1 cells, while the H1299-RESTKO and H1299-RESTKO-PROX1 cells expressed high levels of CHG-A (Fig. 1d). These findings were consistent with the results of previous studies showing that REST is the major regulator of CHG-A expression (D’Alessandro et al. 2008).

### REST deficiency induces and PROX1 accelerates ESG-related gene expression in H1299 cells

**Fig. 2:**
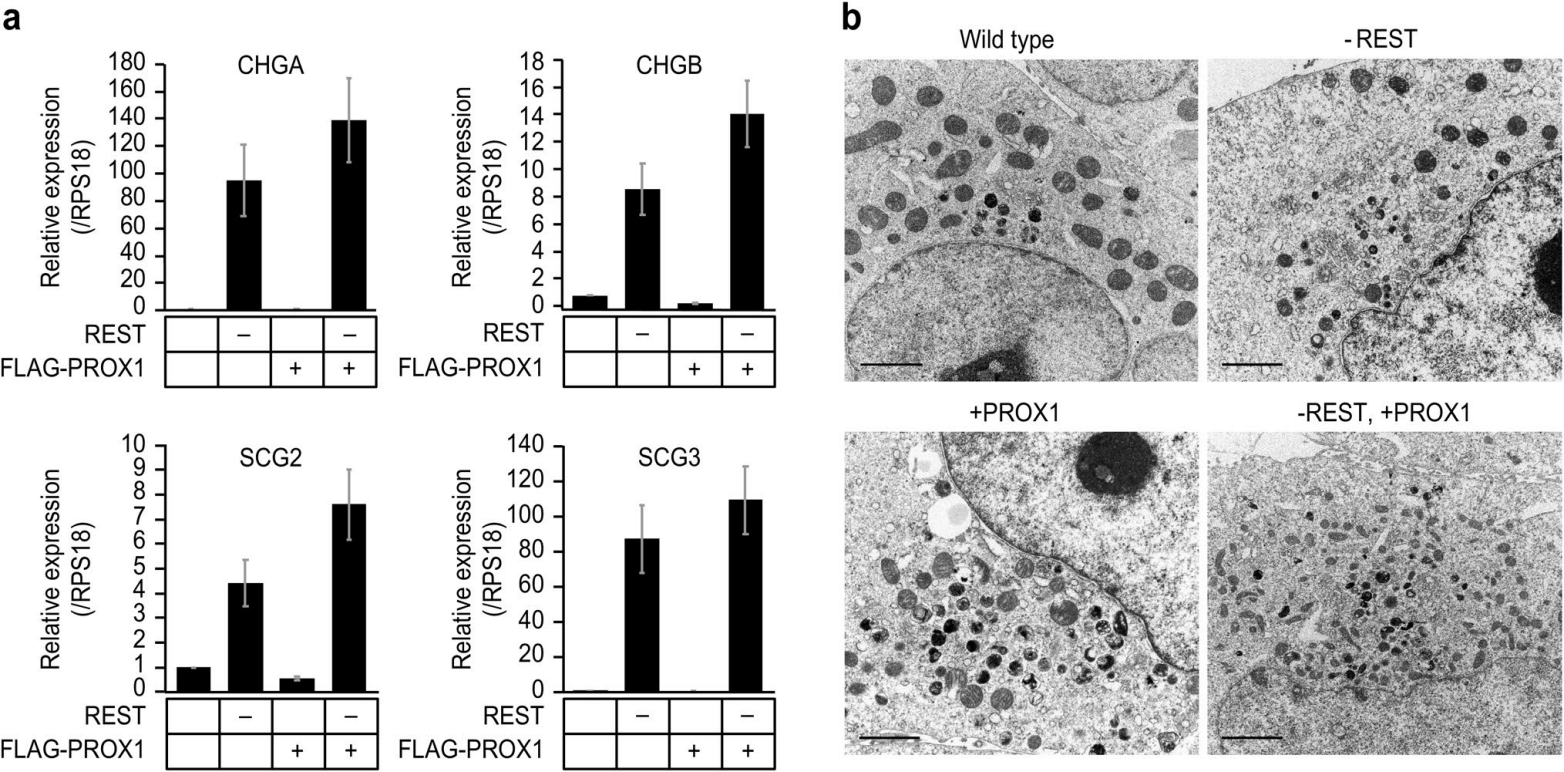
Effects of REST and PROX1 on ESG-related gene expression and ESG formation in H1299 cells. **a** Quantitative RT-PCR analyses were conducted to examine alterations in the expression of ESG-related genes induced by the knockout of *REST* and forced expression of FLAG-PROX1. "−" indicates that a specific gene was knocked out, and "+" indicates that a specific gene was transfected. The expression levels of CHG-A (chromogranin-A), CHG-B (chromogranin-B), SCG-2 (secretogranin-II) and SCG-3 (secretogranin-III) were highly induced by knockout of *REST* (− REST) and enhanced by PROX1 transfection (+ PROX1). Ribosomal protein S18 (RPS18) served as an internal control. The data are shown as the mean ± SD. **b** Transmission electron micrographs of wild-type H1299 cells and gene-transfected/gene-edited H1299 cells. "−" indicates that a specific gene was knocked out, and "+" indicates that a specific gene was transfected. No ESGs were observed in any of the cells. Scale bar, 2 μm

We then investigated the synergistic effects of REST deficiency and PROX1 expression on ESG-related gene expression. The expression of CHG-A, CHG-B, SCG-2, and SCG-3 in the H1299 transfectants was analysed by qRT-PCR (Fig. 2a). CHG-A was highly expressed in the H1299-RESTOKO cells but not in the H1299-PROX1 or wild-type H1299 cells. However, compared with that in the H1299-RESTKO cells, the level of CHG-A was higher (~ 140 times) in the H1299-RESTKO-PROX1 cells, and the same phenomena were observed for other ESG-related genes. These results demonstrated that knockout of *REST* is indispensable for the expression of ESG-related genes in nonendocrine cells and that PROX1 enhances the expression of ESG-related genes. Since the expression of ESG-related genes was markedly elevated in the H1299-RESTKO and H1299-RESTKO-PROX1 cells, we conducted electron microscopic analysis to investigate ESG formation. However, ESG formation was not found in the H1299-RESTKO, H1299-PROX1 or H12299-RESTKO-PROX1 cells (Fig. 2b).

### An additional transgene encoding an intragranular secretory molecule enhances the number of ESGs in the H1299-RESTKO-PROX1 cells

**Fig. 3:**
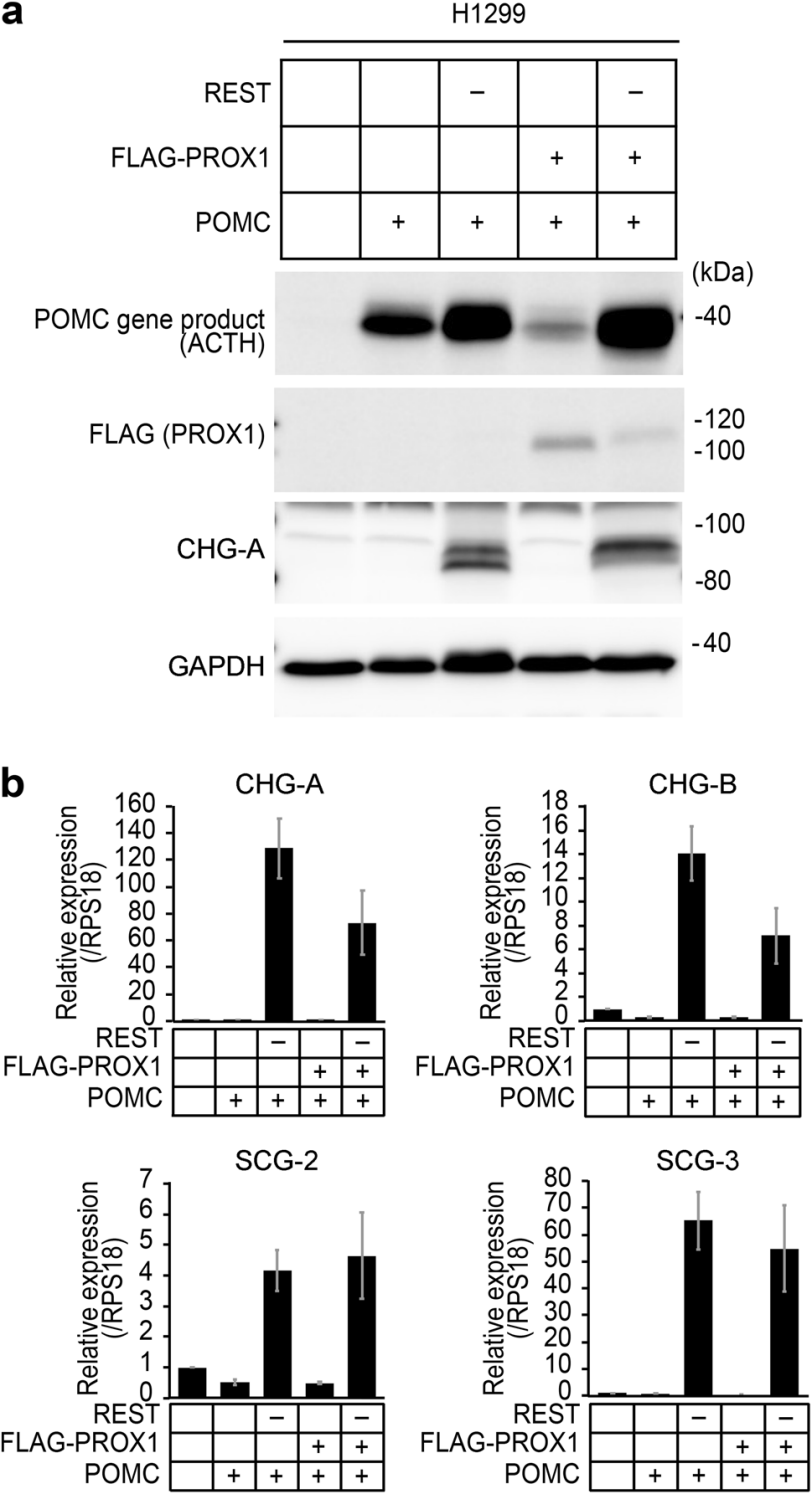
Effects of POMC on ESG-related gene expression in H1299 cells. **a** Western blot analysis of POMC gene products in H1299 transfectants. Signals of POMC gene products were observed in the POMC-transfected H1299 cells. CHG-A was induced in the *REST*-deficient H1299 cells. GAPDH served as an internal control. "+" indicates that a specific gene was transfected, and "−" indicates that a specific gene was knocked out. MW, molecular weight marker. **b** Quantitative RT-PCR analyses were conducted to examine alterations in the expression of ESG-related genes induced by the knockout of REST and forced expression of FLAG-PROX1/POMC. "−" indicates that a specific gene was knocked out, and "+" indicates that a specific gene was transfected. Sustained expression of ESG-related genes (CHG-A, CHG-B, SCG-2 and SCG-3) was found in the − REST H1299 cells and the − REST + PROX1 H1299 cells despite the additional transfection of POMC. Ribosomal protein S18 (RPS18) served as an internal control. The data are shown as the mean ± SD

We speculated why ESG formation did not occur in the H1299-RESTKO-PROX1 cells despite the high expression of ESG-related genes. Since the forced expression of prohormones reportedly induces ESG-like vesicles (Beuret et al. 2004), we hypothesized that intragranular secretory content is necessary for the formation of ESGs. We chose to utilize POMC as the intragranular secretory molecule to assess ESG formation in H1299 cells because small cell lung cancer, a representative malignant lung neuroendocrine cancer, often produces adrenocorticotropic hormone (ACTH), a mature form of POMC, as an ectopic hormone and is known to cause Cushing's syndrome (Perakakis et al. 2011). Then, we transfected POMC cDNA into H1299, H1299-RESTKO, H1299-PROX1, and H1299-RESTKO-PROX1 cells and confirmed the induction of the POMC gene product in the H1299 line and each of the H1299-derived cell lines (Fig. 3a). ESG-related gene expression in the REST-deficient and/or PROX1-expressing H1299 cells was sustained even when POMC was additionally transfected (Fig. 3b).

**Fig. 4:**
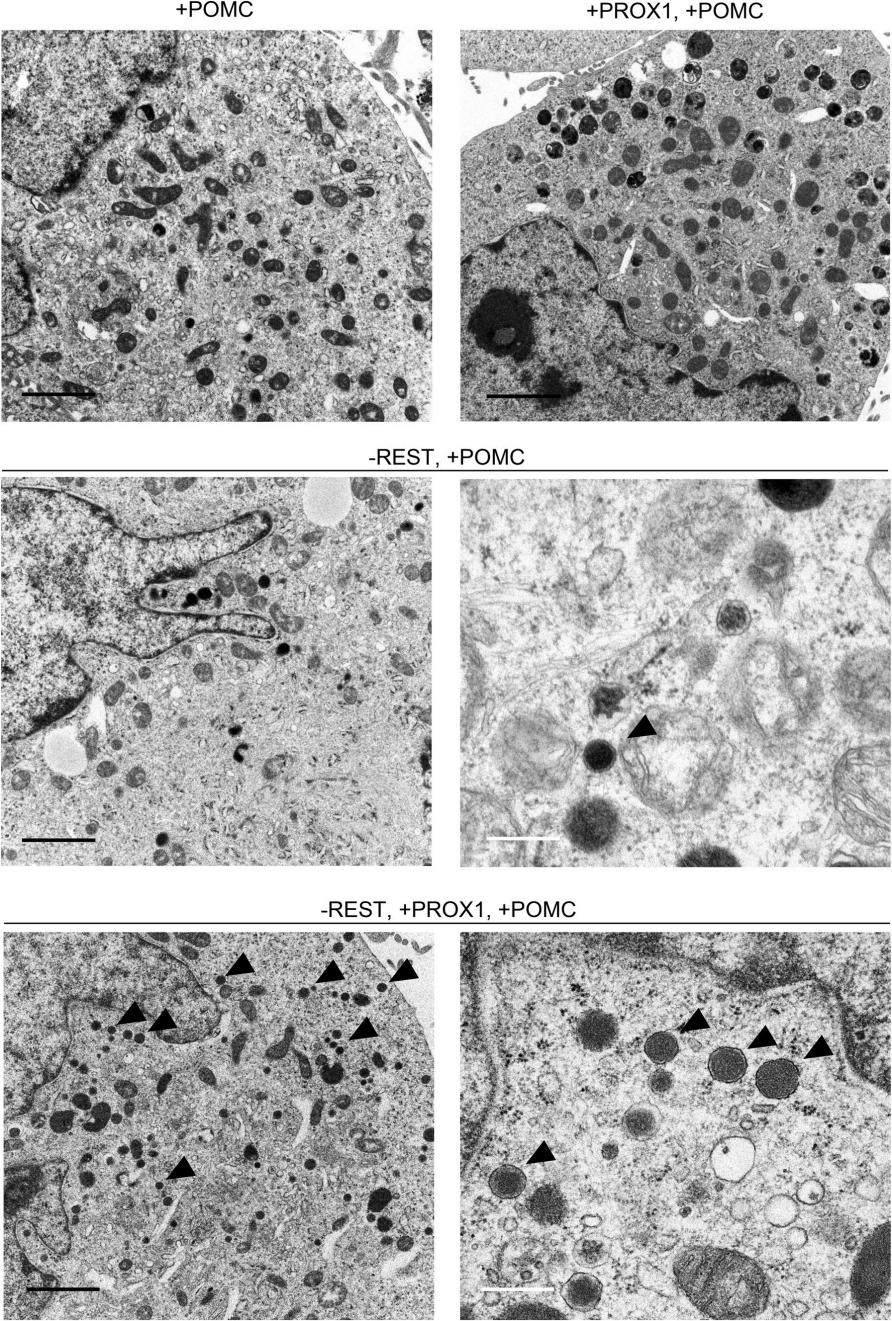
*REST* knockout and PROX1/POMC expression induce ESG-like structures in H1299 cells. Transmission electron micrographs of the gene-transfected/gene-edited H1299 cells. Many ESG-like structures were observed in the − REST + PROX1 + POMC H1299 cells (H1299-RESTKO-PROX1-POMC), and the same structures were observed in the − REST + POMC H1299 cells (H1299-RESTKO-POMC) but to a lesser extent. "+" indicates that a specific gene was transfected, and "−" indicates that a specific gene was knocked out. The arrowheads indicate representative ESG-like structures. Black scale bar, 2 μm. White scale bar, 0.5 μm

Next, we performed electron microscopic analysis again to examine alterations in ESG formation in association with the induction of a gene encoding an intragranular molecule. As shown in Figs. 1a and 2b, no ESGs were observed in the H1299, H1299-RESTKO, H1299-PROX1, or H1299-RESTOKO-PROX1 cells. However, many spherical intracellular structures with unit membranes and highly electron-dense cores were observed in the cytoplasm of the H1299-RESTKO-PROX1-POMC cells (Fig. 4), and the same structures were observed in the H1299-RESTKO-POMC cells but to a lesser extent (Fig. 4). These intracellular structures were 200 to 400 nm in diameter and were structurally similar to ESGs that are usually found in endocrine cells, and they were also found in the vicinity of the Golgi apparatus in the H1299-RESTKO-PROX1-POMC cells (Online Resource 1). These data suggest their suitable quality for ESG biogenesis in nonendocrine cells. Then, we measured the areas of ESG-like structures and lysosomes in each cell line (Online Resource 2). We measured the area of lysosomes because these organelles are used for exocytosis in nonendocrine cells (Dannies 1999) and function in the numerical regulation of ESGs in certain endocrine cells (Schieber et al. 2015). The tendency for lysosomes to decrease in the H1299-RESTKO-PROX1-POMC cells (Online Resource 2) was also shown by immunohistochemistry (Online Resource 3). As a result, the following trends were observed: the area of ESG-like structure was found to be higher in the H1299-RESTKO-PROX1-POMC cells, followed by the H1299-RESTKO-POMC cells. The lysosomal area was decreased by REST knockout in the H1299 cells and was the lowest in the H1299-RESTKO-PROX1-POMC cells.

### Colocalization of CHG-A and POMC gene products in the ESGs of the H1299-RESTKO-PROX1-POMC cells

**Fig. 5:**
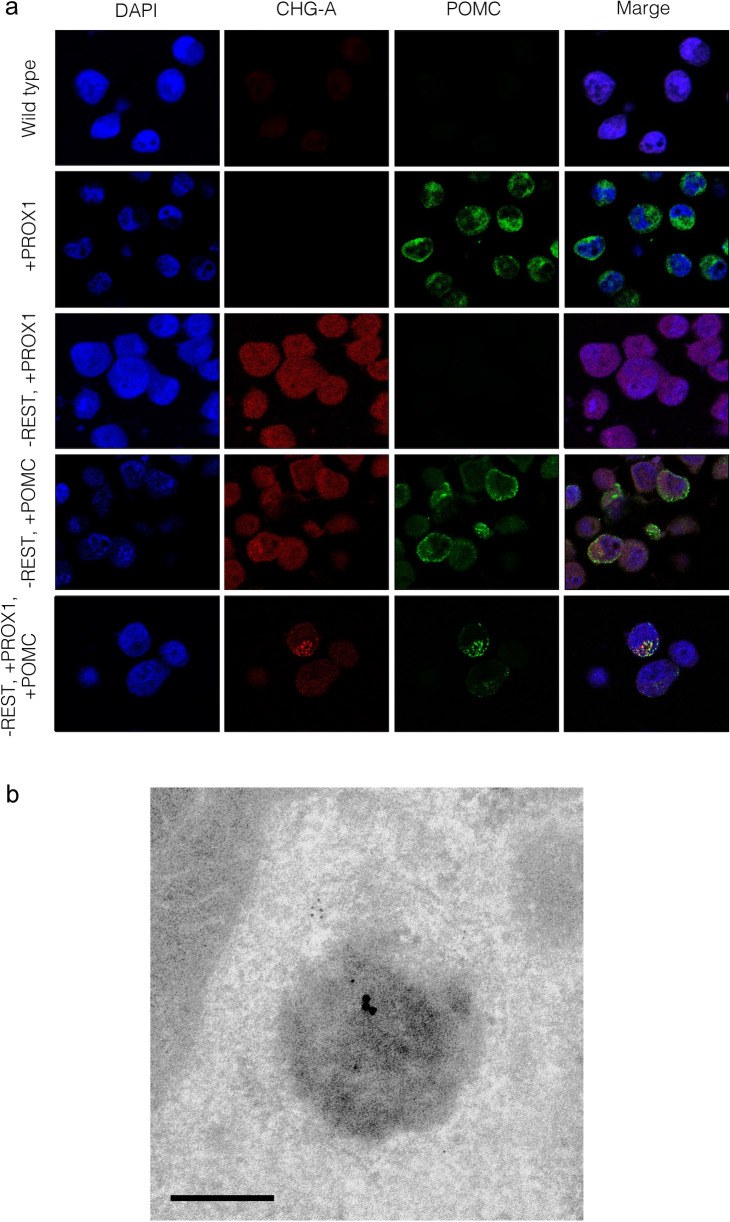
Immunofluorescence cytochemical and immunoelectron microscopic analyses of CHG-A and POMC in the *REST*-deficient H1299 cells transfected with or without PROX1 and/or POMC. **a** H1299 cells (wild-type), REST-deficient PROX1-transfected H1299 cells (− REST + PROX1), REST-deficient POMC-transfected H1299 cells (− REST + POMC), and REST-deficient PROX1- and POMC-transfected H1299 cells (− REST + PROX1 + POMC) were stained with a rabbit polyclonal anti-CHG-A antibody (Alexa Fluor 555-labelled), a mouse monoclonal anti-ACTH antibody (Alexa Fluor 488-labelled), which was used to detect POMC gene products, and DAPI (blue). Images were acquired by a confocal microscope. Red signals (CHG-A) and green signals (POMC) were colocalized in the − REST + PROX1 + POMC H1299 cells (H1299-RESTKO-PROX1-POMC). **b** PROX1- and POMC-transfected REST-deficient H1299 cells (H1299-RESTKO-PROX1-POMC) were processed for immunoelectron microscopy. Thin sections were treated with a rabbit polyclonal anti-CHG-A antibody and a mouse monoclonal anti-ACTH antibody, which was used to detect POMC gene products, and then incubated with an anti-rabbit secondary antibody coupled to 5 nm gold and an anti-mouse secondary antibody coupled to 15 nm gold. Gold colloids sized 5 nm and 15 nm were colocalized on the ESGs. Scale bar, 100 nm

We then performed immunofluorescence analysis to determine whether the CHG-A protein and POMC gene product were colocalized in the REST-deficient H1299 transfectants in which the PROX1, POMC and PROX1/POMC genes were introduced. As a result, CHG-A signals were detected weakly and diffusely in the cytoplasm of the H1299-RESTKO-PROX1 cells, and a green signal indicating the expression of the POMC gene product was observed diffusely or partially in the cytoplasm of the H1299-POMC and H1299-RESTKO-POMC cells. Colocalized granular signals of the CHG-A and POMC gene products were not detected in the H1299-RESTKO-PROX1 cells. However, colocalized signals were observed in the cytoplasm of the H1299-RESTKO-POMC cells and the H1299-RESTKO-PROX1-POMC cells (Fig. 5a). The average number of colocalized signals in one field of view of the fluorescent image was 2.5 for the H1299-RESTKO-POMC cells and 11.3 for the H1299-RESTKO-PROX1-POMC cells. Furthermore, the immunoelectron microscopy results confirmed that the CHG-A and POMC gene products were colocalized in the ESGs produced in the H1299-RESTKO-PROX1-POMC cells. As shown in Fig. 5b, 15 nm gold colloidal particles indicated the presence of CHG-A, and 5 nm gold colloidal particles indicated the colocalization of the POMC gene product in the ESG structures. Although the expression of ESG-related genes was enhanced in the H1299-RESTKO-PROX1 cells in which POMC was not introduced, no POMC gene product signal was observed, but CHG-A signals were scattered in the cytoplasm. No accumulating findings at specific locations were noted in the H1299-RESTKO-PROX1 cells (Online Resource 4).

## Discussion

In this study, we directly confirmed ESG synthesis in the REST-deficient and PROX1- and POMC-expressing non-neuroendocrine cell line H1299 (H1299-RESTKO-PROX1-POMC) by immunofluorescence, electron, and immunoelectron microscopy. The ESGs formed in the H1299-RESTKO-PROX1-POMC cells were 200 to 400 nm in diameter and had unit membranes and dense-core granules. Furthermore, ESG-related and intragranular hormonal molecules colocalized in ESGs. In previous studies, ESG-like structures were presumably formed in nonendocrine cell lines by the expression of CHG-A or CHG-B (Kim et al. 2001; Huh et al. 2003). Forced expression of prohormones such as provasopressin, pro-oxytocin, and POMC induced ESG-like vesicles in the nonendocrine African green monkey renal interstitial cell line COS-1, suggesting that intragranular hormonal molecules act as assembly factors and contribute to the formation of ESGs (Beuret et al. 2004). However, forced expression of the prohormone POMC alone did not induce ESG-like structures in the nonendocrine H1299 cells used in this study. The difference between the previous study and this study might be caused by the difference in the cell lines used. In endocrine cells and certain nonendocrine cells, the conditions necessary for the formation of ESGs are inherently provided, and ESGs are formed only by forced expression of secreted proteins (Chevrier et al. 1991). However, the intracellular environment that enables ESG formation by altering the expression of genes such as REST and PROX1 is necessary for certain nonendocrine cells, including H1299 cells, to form ESG-like structures; therefore, secretory proteins alone are not sufficient to induce ESG-like structures.

We selected a POMC gene product as the secretory protein in this study, but whether the same result can be obtained by the forcible expression of other secretory proteins remains unknown. The shape of ESGs may differ depending on the type of secreted protein, such as insulin, glucagon, and somatostatin, in hormone-producing pancreatic Langerhans cells of the pancreas (Larsson 1978). It is interesting to observe how the type of secretory protein affects the shape of the ESG-like structure. By using the H1299 transfectants established in this study, researchers can perform detailed analyses of the relationships between secretory proteins and the shape of ESGs.

In this study, we used H1299, a non-neuroendocrine cancer (non-NEC) cell line derived from the lung. The lung is a specific organ in which NECs are frequently generated, accounting for approximately 15% of lung cancers (Travis et al. 2004; Fisseler-Eckhoff and Demes 2012; Yazawa 2015). Furthermore, the transformation of lung adenocarcinomas into NECs has recently attracted the attention of researchers as a major mechanism of resistance to gene-targeting therapies (Zakowski et al. 2006; Sequist et al. 2011; Niederst et al. 2015; Oser et al. 2015). Since the mechanisms of ESG formation might be linked to the transformation of lung adenocarcinomas into NECs, it is important to clarify the molecular mechanisms of ESG biogenesis. We and other investigators have demonstrated that many neuronal genes, such as ESG-related molecules, ion channels, neurotransmitter receptors, and neuron-specific cytoskeleton-related genes, are controlled by REST and PROX1 (Roopra et al. 2001; Bruce et al. 2004; Ishii et al. 2016). Our findings indicate that the expression mechanisms of *REST* and/or *PROX1* will help elucidate the transformation of lung adenocarcinomas into NECs.

In this study, we obtained electron microscopic and immunocytochemical findings that ESGs induced in the H1299-RESTKO-PROX1-POMC cells were localized around the Golgi apparatus (Online Resource 1) and that the amounts of lysosomes that contained various kinds of enzymes, including hormone-processing enzymes, tended to decrease in ESG-induced cell lines (Online Resources 2 and 3). Intracellularly expressed secretory proteins are transported to immature granules by budding from the trans-Golgi network, and immature granules then transform into mature secretory granules through a gradual maturation process. After appropriate secretory stimuli in endocrine cells, secretory granules are fused to the cell membrane, and secretory proteins are then released to the outside of the cell (Tooze et al. 2001). When secretory proteins are expressed as prohormones, they must be properly processed into the secretory form by prohormone convertase (Zhou et al. 1999). The regulatory secretory process needs to proceed normally. Cédric et al*.* reported that the VPS41 subunit of the HOPS complex is involved in the regulated secretory process of ESGs (Asensio et al. 2013), and Burns et al. (2021) reported that VPS41 expression requires the exocytosis of insulin from pancreatic β-cells based on the use of the rat insulinoma cell line INS-1 and conditional gene knockout mice. Asadi et al. reported that stathmin-2 (STMN2) regulates glucagon secretion in pancreatic alpha cells (Asadi and Dhanvantari 2020). Consistent with the fact that STMN2 was reported to be a target of REST (Aoki et al. 2012), the expression of STMN2 was approximately tenfold higher in the H1299-RESTKO cells than in the H1299 cells and was upregulated by an additional transgene of POMC (unpublished observations). Although the molecular mechanisms of regulated secretory pathways are still unclear, the results of this study will help elucidate these mechanisms. Further analyses to confirm whether the ESGs formed in the H1299-RESTKO-PROX1-POMC cells are functional (show regulatory processing and exocytosis) are necessary to completely elucidate the regulatory secretory pathways.

In summary, although ESG production was not observed in the REST-deficient and PROX1-expressing H1299 cells, a small number of ESGs were produced in the REST-deficient and POMC-expressing cells, and numerous ESGs were produced in the REST-deficient and PROX1- and POMC-expressing cells. These results suggest that REST deficiency and the expression of genes related to ESG content are necessary for ESG production and that ESG production is accelerated by PROX1.

## Supplementary Information

Below is the link to the electronic supplementary material.

Supplementary file1 (PDF 2757 kb)

Supplementary file2 (PDF 324 kb)

Supplementary file3 (PDF 2811 kb)

Supplementary file4 (PDF 2786 kb)

## Data Availability

The data used to support the findings of this study are available from the corresponding author upon request.
