# Supplementary material for: Endocrine secretory granule production is caused by a lack of REST and intragranular secretory content and accelerated by PROX1
Source: J Mol Histol. 2022 Jan 30;53(2):437–48. doi: 10.1007/s10735-021-10055-5 (PMC9117388; doi:10.1007/s10735-021-10055-5)
Supplement: Supplementary file 1 — Supplementary file1 (PDF 2757 kb) [file 10735_2021_10055_MOESM1_ESM.pdf]

Online Resource 1

ESG-like structures found near the Golgi apparatus

Transmission electron micrographs of the REST-deficient PROX1- and POMC-transfected (－REST+PROX1+POMC) H1299 cells. Some ESG-like structures were identified in the vicinity of the Golgi apparatus. The same image with different magnifications is shown. The arrowheads indicate representative ESG-like structures. N, nucleus. G, Golgi apparatus. Black scale bar, 1 μm. White scale bar, 0.5 μm.

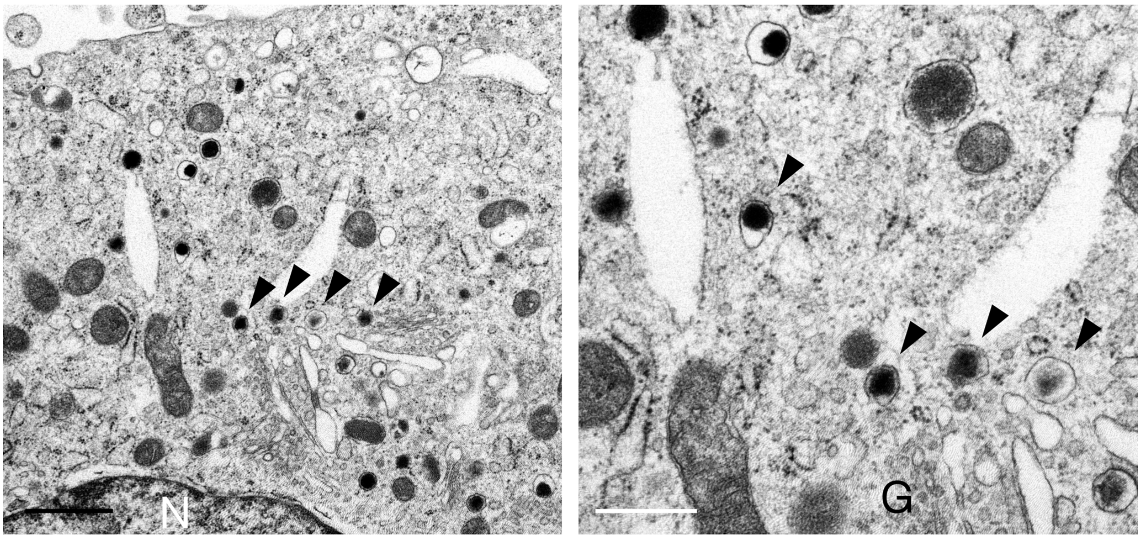

“**Endocrine secretory granule production is caused by a lack of REST and intragranular secretory content and accelerated by PROX1**”,

Journal of Molecular Histology,

Jun Ishii, Hanako Sato-Yazawa, Korehito Kashiwagi, Kazuhiko Nakadate, Masami Iwamoto, Kakeru Kohno, Chie Miyata-Hiramatsu, Meitetsu Masawa, Masato Onozaki, Shuhei Noda, Tadasuke Miyazawa, Megumi Takagi, Takuya Yazawa.

Correspondance to Takuya Yazawa (Dokkyo Medical University School of Medicine and Graduate School of Medicine, Tochigi, Japan, tkyazawa@dokkyomed.ac.jp)
