# Supplementary material for: Endocrine secretory granule production is caused by a lack of REST and intragranular secretory content and accelerated by PROX1
Source: J Mol Histol. 2022 Jan 30;53(2):437–48. doi: 10.1007/s10735-021-10055-5 (PMC9117388; doi:10.1007/s10735-021-10055-5)
Supplement: Supplementary file 2 — Supplementary file2 (PDF 324 kb) [file 10735_2021_10055_MOESM2_ESM.pdf]

Online Resource 2

Measurement of ESG-like structure and lysosome areas in the H1299 cells and the transfectants

Based on the electron micrographs of the H1299 cells and the H1299 cells in which REST was knocked out, PROX1 was introduced and/or POMC was introduced, multiple areas of ESG-like structures (top) and lysosomes (bottom) in one field were measured using the image analysis software ImageJ, and the results were graphed.

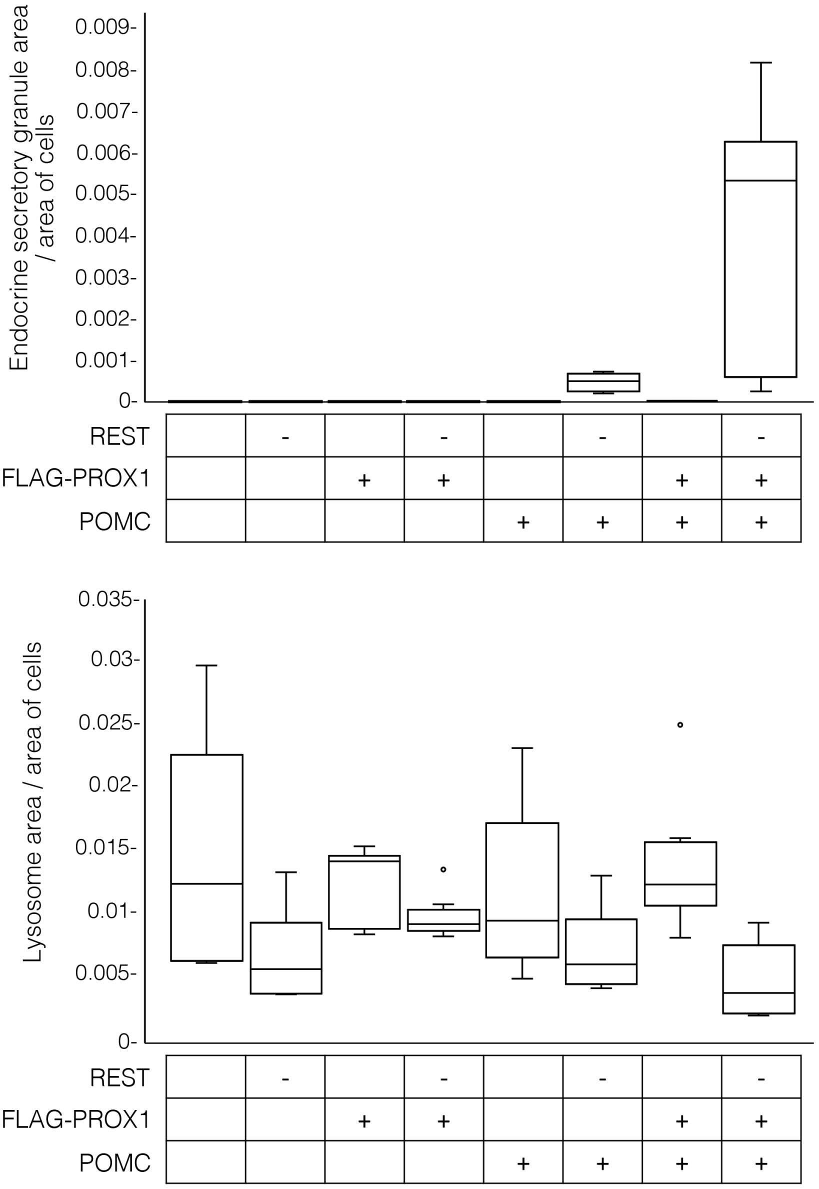

“**Endocrine secretory granule production is caused by a lack of REST and intragranular secretory content and accelerated by PROX1**”,

Journal of Molecular Histology,

Jun Ishii, Hanako Sato-Yazawa, Korehito Kashiwagi, Kazuhiko Nakadate, Masami Iwamoto, Kakeru Kohno, Chie Miyata-Hiramatsu, Meitetsu Masawa, Masato Onozaki, Shuhei Noda, Tadasuke Miyazawa, Megumi Takagi, Takuya Yazawa.

Correspondance to Takuya Yazawa (Dokkyo Medical University School of Medicine and Graduate School of Medicine, Tochigi, Japan, tkyazawa@dokkyomed.ac.jp)
