# Supplementary material for: Endocrine secretory granule production is caused by a lack of REST and intragranular secretory content and accelerated by PROX1
Source: J Mol Histol. 2022 Jan 30;53(2):437–48. doi: 10.1007/s10735-021-10055-5 (PMC9117388; doi:10.1007/s10735-021-10055-5)
Supplement: Supplementary file 3 — Supplementary file3 (PDF 2811 kb) [file 10735_2021_10055_MOESM3_ESM.pdf]

Online Resource 3

Expression of a lysosome marker (lysosome-associated membrane protein 2, LAMP2) in the H1299 and H1299-RESTKO-PROX1-POMC cells

(Top) Immunocytochemical analysis of lysosome-associated membrane protein 2 (LAMP2, 1:1000, GTX103214, GeneTex) in the H1299 cells and the REST-deficient PROX1- and POMC-transfected H1299 cells (H1299-RESTKO-PROX1-POMC).

(Bottom) Quantitative analysis of LAMP2 signals in the H1299 and H1299-RESTKO-PROX1-POMC cells by using ImageJ software. Signals indicating LAMP2 were detected in both cell lines but tended to be lower in the H1299-RESTKO-PROX1-POMC cells.

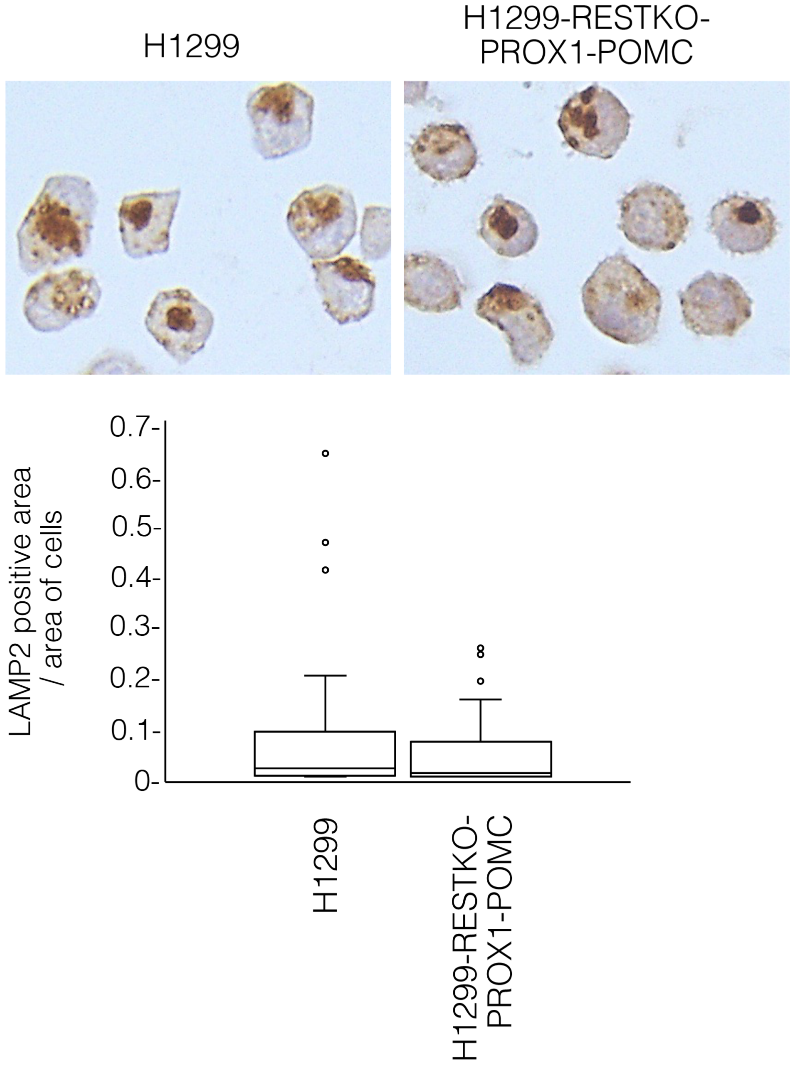

“**Endocrine secretory granule production is caused by a lack of REST and intragranular secretory content and accelerated by PROX1**”,

Journal of Molecular Histology,

Jun Ishii, Hanako Sato-Yazawa, Korehito Kashiwagi, Kazuhiko Nakadate, Masami Iwamoto, Kakeru Kohno, Chie Miyata-Hiramatsu, Meitetsu Masawa, Masato Onozaki, Shuhei Noda, Tadasuke Miyazawa, Megumi Takagi, Takuya Yazawa.

Correspondance to Takuya Yazawa (Dokkyo Medical University School of Medicine and Graduate School of Medicine, Tochigi, Japan, tkyazawa@dokkyomed.ac.jp)
