# Supplementary material for: Endocrine secretory granule production is caused by a lack of REST and intragranular secretory content and accelerated by PROX1
Source: J Mol Histol. 2022 Jan 30;53(2):437–48. doi: 10.1007/s10735-021-10055-5 (PMC9117388; doi:10.1007/s10735-021-10055-5)
Supplement: Supplementary file 4 — Supplementary file4 (PDF 2786 kb) [file 10735_2021_10055_MOESM4_ESM.pdf]

Online Resource 4

Distribution of CHG-A in the H1299-RESTKO-PROX1 cells

Immunoelectron micrography of the REST-deficient and PROX1-transfected H1299 cells. Thin sections were treated with a rabbit polyclonal anti-CHG-A antibody and a mouse monoclonal anti-ACTH antibody, and then incubated with an anti-rabbit secondary antibody coupled to 5 nm gold and an anti-mouse secondary antibody coupled to 15 nm gold. Gold colloids sized 15 nm (ACTH, POMC gene product) were not observed, 5 nm (CHG-A) was scattered in the cytoplasm, and no accumulation in a specific structure was confirmed. Black scale bar, 1 μm. White scale bar, 0.2 μm.

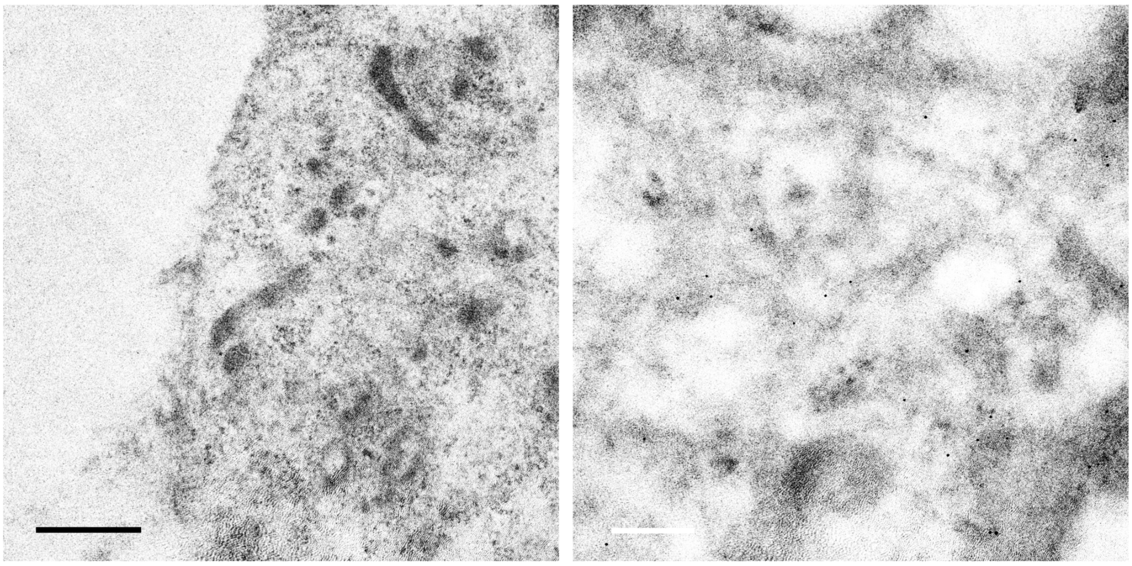

"Endocrine secretory granule production is caused by a lack of REST and intragranular secretory content and accelerated by PROX1",

Journal of Molecular Histology,

Jun Ishii, Hanako Sato-Yazawa, Korehito Kashiwagi, Kazuhiko Nakadate, Masami Iwamoto, Kakeru Kohno, Chie Miyata-Hiramatsu, Meitetsu Masawa, Masato Onozaki, Shuhei Noda, Tadasuke Miyazawa, Megumi Takagi, Takuya Yazawa.

Correspondance to Takuya Yazawa (Dokkyo Medical University School of Medicine and Graduate School of Medicine, Tochigi, Japan, tkyazawa@dokkyomed.ac.jp)
